# Sex Differences in Bone Health Among Indian Older Adults with Obesity, Sarcopenia, and Sarcopenic Obesity

**DOI:** 10.1007/s00223-022-00981-1

**Published:** 2022-05-04

**Authors:** Anoohya Gandham, David Scott, Maxine P. Bonham, Bharati Kulkarni, Sanjay Kinra, Peter R. Ebeling, Ayse Zengin

**Affiliations:** 1 Department of Medicine, School of Clinical Sciences at Monash Health, Monash University, Clayton, VIC 3168 Australia; 2 Institute for Physical Activity and Nutrition (IPAN), School of Exercise and Nutrition Sciences, Deakin University, Geelong, Australia; 3 Department of Nutrition, Dietetics and Food, Monash University, Notting Hill, VIC Australia; 4 Clinical Division, National Institute of Nutrition, Jamai Osmania PO, Hyderabad, India; 5 Department of Non-Communicable Disease Epidemiology, London School of Hygiene & Tropical Medicine, London, UK

**Keywords:** Sarcopenia, Obesity, Older adults, Bone

## Abstract

Age-related changes in fat and lean mass contribute to bone health, but these associations may be influenced by sex and ethnicity. This study investigated sex-specific associations of obesity and sarcopenia with bone mineral density (BMD) and bone mineral apparent density (BMAD) among Indian older adults. 1057 adults aged ≥ 50 years were included. Dual-energy X-ray absorptiometry (DXA) measured BMD at the hip, spine and whole-body, and BMAD was calculated as BMD/√bone area. Obesity was defined by body fat percentage (cut points; > 25% for men and > 35% for women), and sarcopenia was defined using the revised Asian Working Group for Sarcopenia classification with low hand grip strength (< 28 kg for men and < 18 kg for women) and appendicular lean mass index (< 7.0 kg/m^2^ for men and < 5.4 kg/m^2^ for women). Participants were classified into four groups: controls (no obesity or sarcopenia), obesity, sarcopenia, or sarcopenic obesity. Linear regression (*β*-coefficients and 95%CI) analyses were performed with adjustments for age, smoking status, protein intake, and socioeconomic status. Prevalence of sarcopenia (37%) and sarcopenic obesity (6%) were higher in men than women (17% and 4%, respectively). Compared with controls, men with obesity had lower whole-body BMD and BMAD, but women with obesity had higher hip and spine BMD and BMAD (all *p* < 0.05). Men, but not women, with sarcopenic obesity, had lower hip and whole-body BMD and BMAD (all *p* < 0.05) than controls. Men with sarcopenia had lower BMD and BMAD at the hip only, whereas women had lower BMD at all three sites and had lower BMAD at the hip and spine (all *p* < 0.05), compared with controls. Obesity, sarcopenia, and sarcopenic obesity have sex-specific associations with BMD and BMAD in Indian older adults. With the aging population in India, it is important to understand how body composition contributes to poor bone health among older adults.

**Supplementary Information:**

The online version contains supplementary material available at 10.1007/s00223-022-00981-1.

## Introduction

The aging population of India is increasing at an exponential rate, and by 2050, 19% of the total population is predicted to be aged 60 years and over, comprising 324 million individuals [1]. It is estimated that there will be parallel increases in chronic age-related diseases such as osteoporosis, and osteoporosis-related morbidity is likely to increase [2, 3]. Currently, osteoporosis affects 1 in 5 adults aged 18–59 years in India, with the prevalence higher in women compared with men [4, 5]. The onset of osteoporosis occurs approximately 10–20 years earlier in Indians compared with Western populations, highlighting their increased risk for low bone mineral density (BMD) [5]. It is possible that ethnic differences in body composition, dietary patterns, and physical function contribute to this increased prevalence among Indians compared with Western populations [3, 4, 6].

Sarcopenia is another age-associated condition and is defined as the loss of skeletal muscle mass and function which increases the risk of adverse musculoskeletal outcomes [7–10]. The prevalence of sarcopenia for those aged > 45 years is between 5 and 39%, depending on the definition of sarcopenia [11]. Additionally, the prevalence of sarcopenia is reported to be higher among Indian men than in women [11]. Obesity also continues to pose a growing threat to Indians with the prevalence expected to triple among adults aged between 20 and 69 years by 2040, with more women than men affected [12]. Obesity has been reported to have a protective effect on bone health, perhaps due to soft-tissue padding at certain sites [12]. However, studies have reported that older adults with combined sarcopenia and obesity, known as “sarcopenic obesity”, have poorer bone health and physical function [8, 9]. As a result, older adults with sarcopenic obesity have increased risk for morbidity, falls, and fractures [8–10, 13–15]. Most of the studies exploring these relationships have been conducted in Caucasian populations [8, 9, 15]. Recently, a study of 631 adults aged 65 years and older from East China reported older men were more likely to have sarcopenia and sarcopenic obesity compared with women, when defined by the Asian Working Group for Sarcopenia criteria [16]. In addition, it was also reported that older men with sarcopenic obesity were more likely to have osteoporosis and dyslipidaemia, whereas women were more likely to have higher blood glucose, suggesting that there may be sex differences in the prevalence and adverse outcomes of sarcopenic obesity [16]. Likewise, another study with 1089 adults aged 50–79 years reported that older women with sarcopenic obesity had higher BMD at the hip but men had similar BMD compared to those with non-sarcopenic non-obesity [9]. There is therefore a need for sex-specific analyses of the effects of sarcopenia and obesity on bone health.

Given the rapidly aging population in India, with concurrent increases in morbidity and hospitalisation, it is important to understand the prevalence of sarcopenia and sarcopenic obesity [17]. Furthermore, no studies have previously explored the associations of obesity, sarcopenia, sarcopenic obesity with bone health in an Indian population of older adults. To address this knowledge gap, we aimed to determine sex-specific differences in BMD, bone mineral apparent density (BMAD), and the prevalence of osteoporosis, between Indian older adults with sarcopenic obesity, obesity alone, sarcopenia alone, and controls (no sarcopenia and obesity).

## Methods

### Study Design and Participants

Data for participants aged ≥ 50 years from two studies, the Indian Migration Study (IMS) and Andhra Pradesh Children and Parents’ Study (APCAPS) were used for this analysis.

IMS was conducted between 2005 and 2007 and initially established to investigate the effects of rural–urban migration on chronic disease risk in India among those aged between 15 and 76 years [17, 18]. All participants of the Hyderabad arm of the IMS were invited to attend a clinic visit to undergo a DXA scan at the National Institute of Nutrition between January 2009 and December 2010. Ethics approval was received for this study and was approved by the All India Institute of Medical Sciences Ethics committee, the National Institute of Nutrition, India and the London School of Hygiene and Tropical Medicine, UK [18, 19]. All participants provided informed consent.

The APCAPS was originally established in 1987–1990 to study the long-term effects of early-life undernutrition on risks of cardiovascular disease and was comprised of children and their mothers aged between 4 and 84 years [20]. The participants were followed up during multiple time points and DXA was assessed at the second follow-up in 2009–2010. Ethics approval was received for this study and was approved by the National Institute of Nutrition, Hyderabad. All participants provided informed consent [19].

### Questionnaires

Age, smoking status, education, and occupation types were self-reported using questionnaires. The highest level of education that was attained was categorized into four groups: completed graduate education, completed secondary schooling, completed primary schooling, and no education. Likewise, the longest occupation held was categorized into four groups: professional, skilled manual, unskilled manual, and unemployed. A validated, semi-quantitative food frequency questionnaire was administered by an interviewer; protein intake data were used in our analyses [21].

### Anthropometry and Muscle Strength

Height (m) was measured to the nearest 0.1 m using a stadiometer (Seca Leicester height measure (portable), Chasmors, UK). Weight (kg) was measured to the nearest 0.1 kg using an electronic scale (Seca Scales model 599) with headgear, accessories, heavy items of clothing including shoes and socks removed. Body mass index (BMI) (kg/m^2^) was calculated using weight (kg) divided by height (m^2^). Waist and hip circumference were measured to the nearest 0.1 cm using a measuring tape and used to calculate the waist/hip ratio.

Hand grip strength was measured to the nearest kilogram using a pneumatic bulb dynamometer (Lafayette Hand Dynamometer Model 78010 for APCAPS and grip D, Takei, Tokyo, Japan for IMS) [19, 20]. Participants were required to use their maximum force by holding the dynamometer which was measured separately three times in APCAPS and four times in IMS in each arm and the maximum value from the dominant arm was used in the analysis.

### Dual Energy X-Ray Absorptiometry (DXA)

Whole-body scans were performed to measure body composition, including body fat percentage and lean mass using a Hologic DXA (Hologic QDR 4500A, Waltham, MA, USA) [11]. Appendicular lean mass (ALM) was calculated as the sum of lean mass in the arms and legs (kg), and appendicular lean mass index (ALMI) was calculated as ALM divided by height squared (kg/m^2^). DXA also measured BMD (g/cm^2^), bone area (cm), and bone mineral content (BMC, g) at the whole body, total hip and lumbar spine.

BMAD was calculated as BMD/√bone area [22]. The current study utilized BMAD as an additional measure of bone density in order to eliminate any confounding effects of short stature within the current study population of older adults [23, 24]. *T*-scores were calculated using hip BMD. Osteopenia was defined as a *T*-score of − 1 to − 2.5 and osteoporosis as a *T*-score of less than − 2.5 (reference hip BMD: women = 0.901 ± 0.111 g/cm^2^; men = 0.988 ± 0.131 g/cm^2^), as previously recommended [25].

### Sarcopenia and Obesity Definitions

Sarcopenia was defined using the revised Asian Working Group for Sarcopenia (AWGS) definition with low hand grip strength cut points of < 26 kg for men and < 18 kg for women and ALMI cut points of < 7.0 kg/m^2^ for men and < 5.4 kg/m^2^ for women [26]. Obesity was defined as BMI ≥ 25 kg/m^2^, consistent with current guidelines for the Indian population but given BMI may not be representative of adiposity and may therefore underestimate obesity, body fat percentage, was utilized for the main analysis [7, 27–30]. Obesity was defined based on DXA-determined body fat percentage using cut points of > 25% for men and > 35% for women [31, 32]. For this study, participants were classified as having sarcopenic obesity, sarcopenia alone, obesity alone, or as controls (no sarcopenia and obesity) based on the above definitions.

### Statistical Analysis

All data analyses were performed using SPSS Statistics 25 (IBM, NY, USA). Characteristics of participants were reported separately for men and women as mean and standard deviations for continuous variables, or as percentages for categorical variables, according to sarcopenia and obesity groups. Chi-squared tests were performed to test differences in the proportion of older adults with osteoporosis, osteopenia, or normal bone health according to sarcopenia and obesity groups.

Linear regression analyses were performed for all participants and stratified by sex to evaluate differences in BMD and BMAD among each of the four groups (sarcopenic obesity, sarcopenia alone, obesity alone, and controls). Older adults with sarcopenic obesity, obesity alone, and sarcopenia alone were compared with controls, and older adults with sarcopenic obesity were also compared with obesity alone and sarcopenia alone groups. Models were adjusted for confounders including age, protein intake and smoking status (Model 2), with a further adjustment for education and occupation types (Model 3). A sensitivity analysis with further adjustments for waist and hip circumference in addition to the confounders in Model 3 was performed in individuals with obesity compared with controls (data not shown). For all analyses, *p* < 0.05 or 95% confidence intervals not including the null point was considered statistically significant.

## Results

In total, 1057 participants (men = 715, women = 342), with a mean age of 55.5 ± 4.9 years, were included in this study (Table 1). A total of 304 older adults (17% men and 12% women) had obesity defined by body fat percentage (regardless of sarcopenia status) and a total of 376 older adults (29% men and 7% women) had sarcopenia (regardless of obesity status). Using the BMI definition, there were 233 (13% men and 9% women) participants with obesity and 11 (1% men and 0.3% women) with sarcopenic obesity. DXA-determined body fat percentage cut-points classified 247 (13% men and 11% women) participants with obesity alone and 57 (4% men and 1% women) with sarcopenic obesity. Women with obesity had higher body fat percentage (39.5%) compared with men with obesity (28.9%). Both men and women with sarcopenia and sarcopenic obesity were shorter and had lower ALM and BMC at both the hip and spine, compared to those with obesity and controls (Table 1 ).

**Table 1:** Participant characteristics by sarcopenia and/or obesity status

Men (*n* = 715)	Controls (*n* = 277)	Obesity (*n* = 135)	Sarcopenia (*n* = 261)	Sarcopenic obesity (*n* = 42)
Age (years)	54.89 ± 4.03	54.70 ± 4.54	58.11 ± 5.81	58.48 ± 5.87
Weight (kg)	57.99 ± 9.28	73.52 ± 10.01	45.81 ± 6.76	59.87 ± 9.06
Height (m)	1.64 ± 0.06	1.65 ± 0.06	1.60 ± 0.06	1.61 ± 0.08
BMI (kg/m^2^)	21.57 ± 3.13	27.00 ± 3.08	17.83 ± 2.13	22.89 ± 2.57
Waist circumference (cm)	79.88 ± 9.41	95.24 ± 8.32	70.31 ± 7.32	86.42 ± 7.75
Waist/hip ratio	0.94 ± 0.06	0.95 ± 0.06	0.93 ± 0.06	0.94 ± 0.07
Educational attainment (%)				
No education	154 (56%)	30 (22%)	217 (83%)	22 (52%)
Primary schooling	57 (21%)	28 (21%)	29 (11%)	8 (19%)
Secondary schooling	57 (21%)	63 (47%)	14 (5%)	9 (21%)
Graduate education	9 (3%)	14 (10%)	1 (0.4%)	3 (7%)
Occupation types (%)				
Unemployed	8 (3%)	15 (11%)	26 (10%)	8 (19%)
Unskilled manual	4 (2%)	3 (2%)	4 (2%)	2 (5%)
Skilled manual	219 (79%)	86 (64%)	215 (82%)	24 (57%)
Professional	46 (17%)	31 (23%)	16 (6%)	8 (19%)
BMC (g)				
Hip	36.10 ± 5.32	35.57 ± 5.17	31.89 ± 5.17	31.14 ± 5.09
Spine	55.14 ± 14.68	55.27 ± 12.17	54.61 ± 17.96	50.16 ± 11.56
BMD (g/cm^2^)				
Hip	0.936 ± 0.115	0.945 ± 0.108	0.846 ± 0.120	0.852 ± 0.098
Spine	0.949 ± 0.197	0.952 ± 0.165	0.960 ± 0.170	0.887 ± 0.165
Body composition				
ALM (kg)	19.71 ± 2.49	21.42 ± 2.94	14.77 ± 2.71	16.70 ± 2.22
Body fat (%)	18.89 ± 4.36	28.94 ± 3.00	16.44 ± 4.21	28.46 ± 3.12

Women (*n* = 342)	Controls (*n* = 157)	Obesity (*n* = 112)	Sarcopenia (*n* = 58)	Sarcopenic obesity (*n* = 15)
Age (years)	53.83 ± 3.66	53.70 ± 3.15	55.10 ± 4.50	55.96 ± 6.01
Weight (kg)	47.39 ± 7.40	63.23 ± 11.14	37.97 ± 4.28	48.72 ± 6.56
Height (m)	1.50 ± 0.05	1.51 ± 0.06	1.47 ± 0.05	1.46 ± 0.06
BMI (kg/m^2^)	20.94 ± 2.89	27.46 ± 3.94	17.58 ± 1.87	22.99 ± 2.79
Waist circumference (cm)	71.70 ± 8.24	87.43 ± 9.38	62.66 ± 5.86	78.80 ± 9.82
Waist/hip ratio	0.83 ± 0.07	0.85 ± 0.06	0.75 ± 0.04	0.86 ± 0.11
Education (%)				
No education	148 (94%)	62 (55%)	58 (100%)	14 (93%)
Primary schooling	6 (4%)	20 (18%)	0 (0%)	1 (7%)
Secondary schooling	3 (2%)	21 (19%)	0 (0%)	0 (0%)
Graduate education	0 (0%)	9 (8%)	0 (0%)	0 (0%)
Occupation types (%)				
Unemployed	31 (20%)	61 (54%)	11 (19%)	7 (47%)
Unskilled manual	8 (5%)	5 (4%)	2 (3%)	0 (0%)
Skilled manual	116 (74%)	35 (31%)	43 (74%)	7 (47%)
Professional	2 (1%)	11 (10%)	2 (3%)	1 (7%)
BMC (g)				
Hip	23.21 ± 4.18	24.82 ± 4.82	19.84 ± 3.45	20.00 ± 1.97
Spine	35.96 ± 8.82	39.54 ± 8.43	31.30 ± 8.27	33.17 ± 6.18
BMD (g/cm^2^)				
Hip	0.758 ± 0.111	0.826 ± 0.126	0.670 ± 0.098	0.713 ± 0.060
Spine	0.745 ± 0.147	0.811 ± 0.141	0.673 ± 0.151	0.714 ± 0.109
Body composition				
ALM (kg)	13.17 ± 1.65	14.65 ± 2.48	10.58 ± 1.01	10.47 ± 1.13
Body fat (%)	29.6 ± 4.36	39.58 ± 3.11	27.46 ± 4.44	39.03 ± 3.27

Data presented as mean ± standard deviation for older men and women

*BMI* body mass index; *BMC* bone mineral content; *BMD* bone mineral density; *ALM* appendicular lean mass

**Fig. 1:**
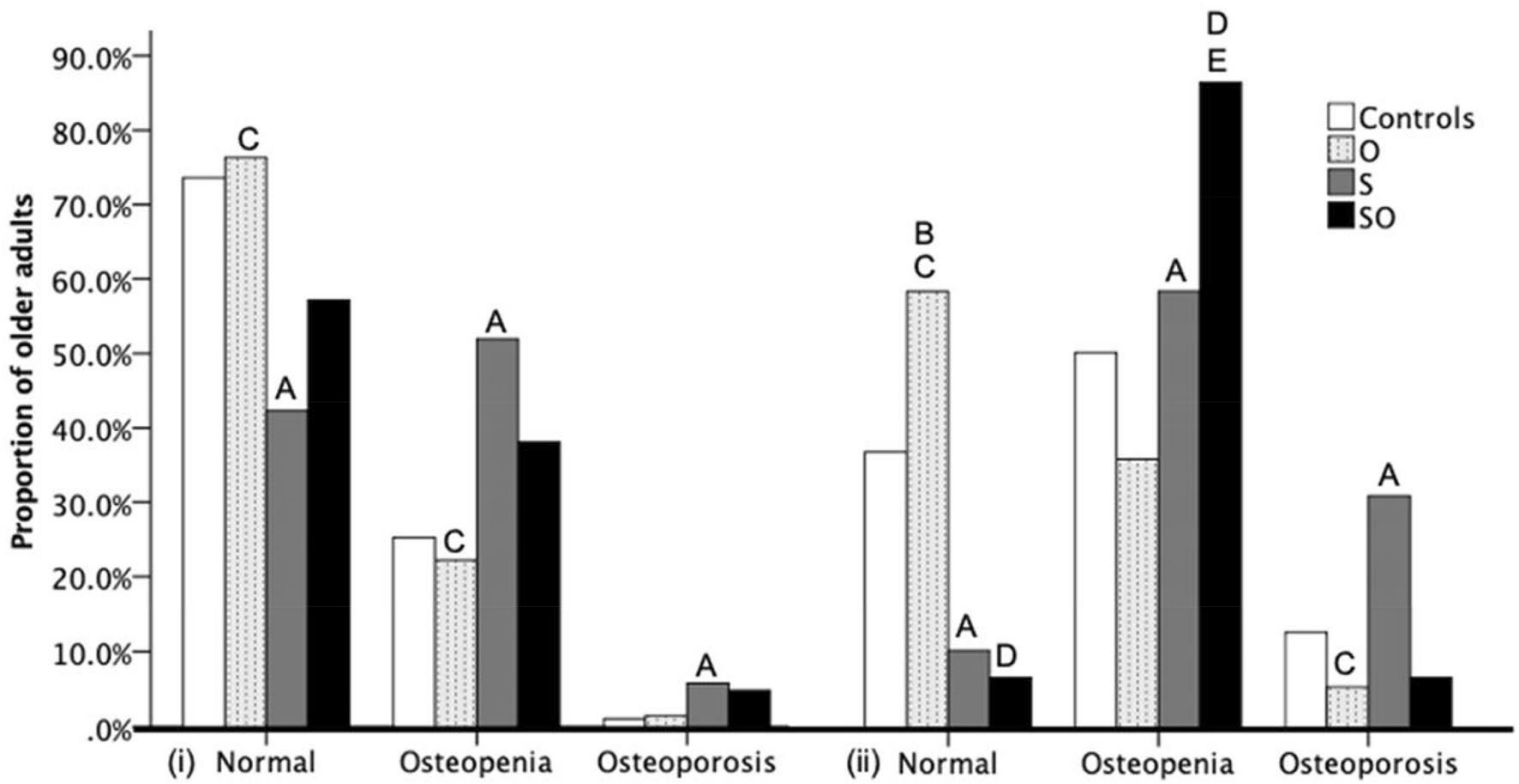
Proportion of older men (i) and women (ii) with osteoporosis, osteopenia, or normal bone health at the hip based on sarcopenia/obesity groups. Significance is denoted by (A) for significant difference between controls and S, (B) for significant difference between controls and O, (C) for significant difference between O and S, (D) for significant difference between O and SO (E) for significant difference between controls and SO. *O* obesity; *S* sarcopenia; *SO* sarcopenic obesity

A higher proportion of men with sarcopenia had osteoporosis at the hip compared with controls. Similarly, a higher proportion of men with sarcopenia had osteopenia compared to both controls and those with obesity. A higher proportion of women with sarcopenia had osteoporosis compared with both control and obesity groups. Women with sarcopenia were also more likely to have osteopenia than controls. A higher proportion of women with sarcopenic obesity had osteopenia compared with both controls and obesity groups (Fig. 1).

Men with obesity had lower whole-body BMD and BMAD, respectively, compared with controls; in contrast, women with obesity had higher BMD and BMAD at the hip and spine (all *p* < 0.05) after adjusting for all confounders (Table 2; model 3). Further adjustment for waist and hip circumference attenuated the associations in women with obesity compared with controls (all *p* > 0.05). Men with sarcopenia had lower hip and whole-body BMD and BMAD compared with controls (model 3). Similarly, women with sarcopenia also had lower hip, spine and whole-body BMD, and lower hip and spine BMAD (all *p* < 0.05, model 3). Following adjustments, men with sarcopenic obesity had lower BMD and BMAD at the hip and whole-body (all *p* < 0.05) compared with controls, while there were no significant differences in women in these groups. Men with sarcopenic obesity had lower whole-body BMD and BMAD compared with those with sarcopenia after adjusting for all confounders (all *p* < 0.05). Likewise, in the total group (men and women combined), those with sarcopenia and sarcopenic obesity had lower hip and whole-body BMD and BMAD compared with controls (Supplementary Table 1). Individuals with obesity had higher hip BMAD but lower whole-body BMD and BMAD compared with controls (all *p* < 0.05).

**Table 2:** Linear regression analyses for comparison of BMD and BMAD at the hip, spine, and whole-body among sarcopenia and obesity groups

Men	Obesity vs *controls*	Sarcopenia vs *controls*	Sarcopenic obesity vs *controls*	Sarcopenic obesity vs *obesity*	Sarcopenic obesity vs *sarcopenia*
Hip BMD					
Model 1	0.009 (− 0.015, 0.033)	**− 0.090 (− 0.110, − 0.071)**	**− 0.084 (− 0.122, − 0.047)**	**− 0.093 (− 0.133, − 0.054)**	0.006 (− 0.031, 0.043)
Model 2	− 0.003 (− 0.027, 0.020)	**− 0.069 (− 0.089, − 0.049)**	**− 0.072 (− 0.109, − 0.036)**	**− 0.069 (− 0.109, − 0.030)**	− 0.004 (− 0.040, 0.033)
Model 3	− 0.003 (− 0.027, 0.021)	**− 0.068 (− 0.089, − 0.048)**	**− 0.072 (− 0.109, − 0.035)**	**− 0.069 (− 0.109, − 0.030)**	− 0.004 (− 0.040, 0.033)
Spine BMD					
Model 1	− 0.007 (− 0.048, 0.033)	− 0.015 (− 0.048, 0.019)	**− 0.072 (− 0.136, − 0.009)**	− 0.065 (− 0.133, 0.003)	− 0.058 (− 0.122, 0.006)
Model 2	− 0.011 (− 0.052, 0.030)	− 0.018 (− 0.054, 0.017)	**− 0.080 (− 0.145, − 0.015)**	− 0.069 (− 0.138, 0.000)	− 0.062 (− 0.126, 0.003)
Model 3	− 0.006 (− 0.049, 0.037)	− 0.022 (− 0.058, 0.013)	**− 0.079 (− 0.144, − 0.015)**	**− 0.074 (− 0.143, − 0.004)**	− 0.057 (− 0.122, 0.008)
Whole-body BMD					
Model 1	**− 0.061 (− 0.082, − 0.039)**	**− 0.026 (− 0.044, − 0.008)**	**− 0.088 (− 0.122, − 0.054)**	− 0.027 (− 0.063, 0.009)	**− 0.061 (− 0.096, − 0.027)**
Model 2	**− 0.0631(− 0.083, − 0.039)**	**− 0.026 (− 0.045, − 0.007)**	**− 0.088 (− 0.123, − 0.054)**	− 0.025 (− 0.061, 0.012)	**− 0.062 (− 0.059, − 0.011)**
Model 3	**− 0.055 (− 0.078, − 0.032)**	**− 0.029 (− 0.048, − 0.011)**	**− 0.086 (− 0.121, − 0.052)**	− 0.027 (− 0.064, 0.010)	**− 0.057 (− 0.091, − 0.022)**
Hip BMAD					
Model 1	0.001 (− 0.003, 0.006)	**− 0.013 (− 0.017, − 0.009)**	**− 0.010 (− 0.017, − 0.003)**	**− 0.011 (− 0.018, − 0.003)**	0.003 (− 0.004, 0.010)
Model 2	0.000 (− 0.005, 0.004)	**− 0.010 (− 0.014, − 0.006)**	**− 0.008 (− 0.015, − 0.001)**	− 0.007 (− 0.015, 0.000)	0.002 (− 0.005, 0.009)
Model 3	− 0.001 (− 0.006, 0.004)	**− 0.010 (− 0.013, − 0.006)**	**− 0.008 (− 0.015, − 0.001)**	− 0.007 (− 0.014, 0.001)	0.002 (− 0.005, 0.009)
Spine BMAD					
Model 1	0.000 (− 0.005, 0.005)	− 0.001 (− 0.005, 0.003)	− 0.007 (− 0.015, 0.001)	− 0.007 (− 0.015, 0.001)	− 0.006 (− 0.014, 0.002)
Model 2	− 0.001 (− 0.006, 0.004)	− 0.001 (− 0.006, 0.003)	− 0.008 (− 0.016, 0.000)	− 0.007 (− 0.016, 0.001)	− 0.007 (− 0.014, 0.001)
Model 3	0.000 (− 0.005, 0.005)	− 0.002 (− 0.006, 0.003)	− 0.008 (− 0.016, 0.000)	− 0.008 (− 0.016, 0.001)	− 0.006 (− 0.014, 0.002)
Whole-body BMAD					
Model 1	**− 0.002 (− 0.002, − 0.001)**	**0.001 (0.000, 0.001)**	**− 0.001 (− 0.002, − 0.001)**	0.000 (0.000, 0.001)	**− 0.002 (− 0.003, − 0.001)**
Model 2	**− 0.002 (− 0.002, − 0.001)**	**0.000 (0.000, 0.001)**	**− 0.001 (− 0.002, − 0.001)**	0.000 (− 0.000, 0.001)	**− 0.002 (− 0.003, − 0.002)**
Model 3	**− 0.001 (− 0.002, − 0.001)**	**0.000 (0.000, 0.001)**	**− 0.001 (− 0.002, − 0.001)**	0.000 (− 0.001, 0.001)	**− 0.002 (− 0.002, − 0.001)**

Women	Obesity vs *controls*	Sarcopenia vs *controls*	Sarcopenic obesity vs *controls*	Sarcopenic obesity vs *obesity*	Sarcopenic obesity vs *sarcopenia*
Hip BMD					
Model 1	**0.068 (0.041, 0.095)**	**− 0.089 (− 0.122, − 0.055)**	− 0.046 (− 0.105, 0.014)	**− 0.114 (− 0.174, − 0.053)**	0.043 (− 0.021, 0.106)
Model 2	**0.058 (0.031, 0.085)**	**− 0.074 (− 0.107, − 0.041)**	− 0.033 (− 0.091, 0.024)	**− 0.091 (− 0.150, − 0.033)**	0.041 (− 0.021, 0.102)
Model 3	**0.055 (0.025, 0.085)**	**− 0.075 (− 0.108, − 0.042)**	− 0.027 (− 0.085, 0.030)	**− 0.082 (− 0.142, − 0.023)**	0.048 (− 0.013, 0.109)
Spine BMD					
Model 1	**0.067 (0.032, 0.102)**	**− 0.072 (− 0.115, − 0.028)**	− 0.031 (− 0.107, 0.045)	**− 0.098 (− 0.175, − 0.021)**	0.041 (− 0.041, 0.122)
Model 2	**0.063 (0.027, 0.098)**	**− 0.060 (− 0.103, − 0.016)**	− 0.017 (− 0.093, 0.058)	**− 0.080 (− 0.157, − 0.003)**	0.042 (− 0.039, 0.123)
Model 3	**0.065 (0.026, 0.105)**	**− 0.060 (− 0.104, − 0.017)**	− 0.013 (− 0.088, 0.062)	− 0.078 (− 0.156, 0.000)	0.047 (− 0.034, 0.128)
Whole-body BMD					
Model 1	− 0.041 (− 0.083, 0.000)	**− 0.075 (− 0.127, − 0.024)**	**− 0.101 (− 0.192, − 0.011)**	− 0.060 (− 0.152, 0.032)	− 0.026 (− 0.123, 0.071)
Model 2	− 0.036 (− 0.078, 0.007)	**− 0.069 (− 0.121, − 0.018)**	− 0.082 (− 0.172, 0.008)	− 0.042 (− 0.134, 0.049)	− 0.013 (− 0.109, 0.084)
Model 3	− 0.019 (− 0.066, 0.028)	**− 0.070 (− 0.122, − 0.019)**	− 0.074 (− 0.164, 0.016)	− 0.046 (− 0.138, 0.045)	− 0.004 (− 0.100, 0.092)
Hip BMAD					
Model 1	**0.014 (0.009, 0.019)**	**− 0.014 (− 0.020, − 0.008)**	− 0.003 (− 0.013, 0.008)	**− 0.016 (− 0.027, − 0.005)**	0.011 (0.000, 0.023)
Model 2	**0.011 (0.006, 0.016)**	**− 0.011 (− 0.017, − 0.005)**	− 0.001 (− 0.011, 0.010)	**− 0.012 (− 0.023, − 0.001)**	0.011 (0.000, 0.022)
Model 3	**0.011 (0.005, 0.016)**	**− 0.012 (− 0.018, − 0.006)**	0.000 (− 0.010, 0.011)	− 0.010 (− 0.021, 0.001)	**0.012 (0.001, 0.023)**
Spine BMAD					
Model 1	**0.009 (0.004, 0.014)**	**− 0.008 (− 0.014, − 0.002)**	− 0.002 (− 0.013, 0.008)	**− 0.012 (− 0.022, − 0.001)**	0.006 (− 0.005, 0.017)
Model 2	**0.009 (0.004, 0.014)**	**− 0.007 (− 0.013, − 0.001)**	− 0.001 (− 0.011, 0.010)	− 0.009 (− 0.020, 0.001)	0.006 (− 0.005, 0.018)
Model 3	**0.009 (0.003, 0.014)**	**− 0.007 (− 0.013, − 0.001)**	0.000 (− 0.010, 0.011)	− 0.009 (− 0.020, 0.002)	0.007 (− 0.004, 0.019)
Whole-body BMAD					
Model 1	**− 0.002 (− 0.003, − 0.001)**	− 0.001 (− 0.002, 0.000)	− 0.002 (− 0.004, 0.000)	0.000 (− 0.002, 0.002)	0.000 (− 0.003, 0.002)
Model 2	**− 0.002 (− 0.003, 0.001)**	− 0.001 (− 0.002, 0.000)	− 0.001 (− 0.004, 0.001)	0.000 (− 0.002, 0.002)	0.000 (− 0.003, 0.002)
Model 3	**− 0.001 (− 0.002, − 0.000)**	− 0.001 (− 0.002, 0.000)	− 0.001 (− 0.003, 0.001)	0.000 (− 0.002, 0.002)	0.000 (− 0.002, 0.002)

Data presented as *β*-coefficients and 95% confidence intervals. Bold indicated *p* < 0.05

Model 1: Unadjusted model

Model 2: Adjusted for confounders including age, smoking status and protein intake

Model 3: Adjusted for confounders in model 2 and socioeconomic status including education and occupation levels

*BMD* bone mineral density; *BMAD* bone mineral apparent density

## Discussion

In this population of Indian older adults, men had a higher prevalence of sarcopenia and sarcopenic obesity compared with women. Sarcopenia was negatively associated with bone health in both men and women, while obesity was positively associated with bone health in women only. Men, but not women, with sarcopenic obesity had worse bone health than controls. These results suggest that there are sex-specific associations of body composition with bone health in older adults, and particularly, obesity does not offset the negative effects of sarcopenia on bone health in older men. In general, the effects on BMD and BMAD were more pronounced in Indian older adults with sarcopenia and sarcopenic obesity.

Sarcopenia and sarcopenic obesity have been previously associated with poor bone health and increased risk of fracture and there may be sex-specific differences in these associations [15, 16]. The current study is the first we are aware of to explore these associations in Indian older adults. We observed that men with sarcopenic obesity had poorer BMD at the hip, spine, and whole-body compared with controls. Similarly, a cross-sectional study in 213 men and 418 women from East China reported that older men, but not women, with sarcopenic obesity were more likely to have osteoporosis and poorer BMD at the hip and spine compared to those with obesity and those with neither sarcopenia nor obesity [16].

Obesity has been considered to have a protective effect on bone health and osteoporosis [33]. In the current study, older women with obesity had higher BMD and BMAD at the hip and spine compared with controls. In contrast, men who were obese had worse BMD and BMAD at the whole-body. There are established sex differences in body fat distribution where men have more visceral fat than subcutaneous fat [34]. In this study, as a surrogate measure of site-specific adiposity, we adjusted for waist and hip circumference and showed that the differences were attenuated in women but not men. Among men, fat may mainly be distributed in the trunk, but women may have a greater distribution in the limbs and hip region [34, 35]. Women may therefore have a greater absorption of impact forces by soft-tissue padding around the hip which may translate to a bone-protective effect [34, 35]. Another possible explanation could be that higher adiposity increases circulating estrogen levels in women and decreases testosterone levels in men [36, 37]. Currently there are no data on sex hormones, body composition, and bone health in Indian adults, and this warrants further investigation [36, 37]. Conflicting with the results of the current study, a recent meta-analysis among ethnically diverse adults reported that obesity may be more strongly positively associated with BMD in men than women [38]. These contradictory findings in the Indian population could be explained by ethnic differences in body composition, physical activity, nutrition, menopausal status, and hormone levels [38, 39]. It is also possible that although individuals with obesity may have increased areal BMD (aBMD) assessed by DXA, they may have compromised bone microarchitecture and strength which cannot be determined from DXA as it is limited to two-dimensional measures of aBMD [7]. Recent findings have also shown that excess soft tissue in those with obesity leads to inaccuracies in DXA-determined aBMD as the absorption of photons by the excess presence of fat around the bone causes a spuriously high reading of aBMD [40, 41]. Future studies should therefore utilize advanced bone imaging modalities such as high-resolution peripheral quantitative computed tomography (HR-pQCT) to investigate the underlying mechanisms between bone microarchitecture and obesity in order to better understand this relationship in Indian older adults [7, 42]. Additionally, these sex-specific associations may be due to differences in the proportion of men (*n* = 715) and women (*n* = 342) in this study. Together, these data suggest that high adiposity in men with sarcopenia does not provide the same beneficial effects on bone health seen in women.

Recent studies have demonstrated a higher prevalence of sarcopenia and sarcopenic obesity among men compared with women in Asian populations [16, 43]. Similarly, in the current study, men had a higher prevalence of sarcopenia and sarcopenic obesity compared with women. Although men usually have higher muscle mass and strength due to differences in sex hormones and body composition, the onset of muscle deterioration may occur much earlier and the magnitude of decline in muscle mass is greater compared with women, therefore increasing their risk for sarcopenia and sarcopenic obesity [16, 43, 44]. Future studies should focus on evaluating the underlying mechanisms contributing to sex differences in the prevalence of sarcopenia and sarcopenic obesity within diverse populations.

The observed prevalence of sarcopenic obesity differed based on the definition of obesity used. When obesity was defined by BMI, only 1% of the population had sarcopenic obesity as opposed to 5% when defined by body fat percentage. A recent study in 1640 children from north India found that BMI misclassified 13–14% boys and 11–14.5% girls into an incorrect adiposity category and therefore recommended the use of body fat percentage to define obesity [45]. Similarly, in a study of 1217 Vietnamese individuals, BMI was found to underestimate the prevalence of obesity and it was concluded that the use of body fat percentage might be a more accurate indicator of obesity status [46]. The underestimation of obesity status by BMI is likely explained by the fact that those with high BMI may often have greater lean mass than those with low BMI. Those with high BMI may therefore be less likely to have concomitant sarcopenia as opposed to when obesity is defined by body fat percentage [28, 29]. In a study of 804 community-dwelling healthy Indian adults, body fat percentage was reported to be a better diagnostic criterion for sarcopenic obesity as it assesses body fat distribution unlike other methods such as by BMI or waist circumference [10]. Although consensus definitions of sarcopenic obesity are not currently available, it would appear that the obesity component should be defined where possible using direct estimates of adiposity such as by DXA.

The strengths of this study includes a well-characterized population from a large cohort of Indian older adults, with direct measurements of body composition and bone density. There are some limitations to this study. Firstly, the proportion of women (32%) in this study was much lower compared with men (68%). This difference in proportions may possibly explain the lack of associations between bone health and sarcopenic obesity in women, as low numbers of women were classified with sarcopenic obesity (*n* = 15). All study participants resided in Hyderabad, India and the results may not be generalized to other regions of India, where there are established differences in lifestyle, and environmental factors [38]. It is also possible for shifts in urbanization and lifestyle habits over time and these findings may therefore not be representative of the current population at the present time. Other confounders which were not assessed in these studies, including physical activity measured with the gold-standard triaxial accelerometer, could have influenced the associations between obesity, sarcopenia, sarcopenic obesity, and bone health. Two different dynamometers were used to measure handgrip strength in the two studies which may have introduced variation. The current study utilized DXA-derived bone parameters and an additional computed measure, BMAD as a measure of bone health. In addition to DXA, the use of other bone imaging devices to measure bone microarchitecture such as HR-pQCT may provide better insights in understanding the underlying relationship between obesity and bone health. Hand grip strength was the only measure of physical function. Other assessments of physical function such as gait speed, chair stand rise, stair climb test, or timed up and go test would be useful as it may contribute to further understanding the differences in poor bone health. Finally, since there is no consensus definition for sarcopenia, the current study utilized the revised AWGS definition as it is the recommended criteria in Asian populations [26]. It should be noted that the current study utilized the AWGS cut points for hand grip strength even though different dynamometers were utilized in this study as AWGS did not propose dynamometer-specific cut points and may therefore not be comparable with other studies [47]. Also, the use of different sarcopenia definitions could influence both prevalence and associations.

In conclusion, in Indian older adults, there are sex-specific associations between obesity, sarcopenia, and sarcopenic obesity with bone health. Men with sarcopenic obesity have worse bone health, but both men and women with sarcopenia had poorer bone health compared with those without sarcopenia and obesity. Obesity may be associated with better bone health in women but not men. Future studies should investigate how sex differences in body composition contributes to poor bone health and determine how they can reduce the risk of falls and fractures among Indian older adults.

## Supplementary Information

Below is the link to the electronic supplementary material.

Supplementary file1 (DOCX 17 kb)
