# Supplementary material for: Sex Differences in Bone Health Among Indian Older Adults with Obesity, Sarcopenia, and Sarcopenic Obesity
Source: Calcif Tissue Int. 2022 May 4;111(2):152–61. doi: 10.1007/s00223-022-00981-1 (PMC9300534; doi:10.1007/s00223-022-00981-1)
Supplement: Supplementary file 1 — Supplementary file1 (DOCX 17 kb) [file 223_2022_981_MOESM1_ESM.docx]

**Supplementary Table 1.** Linear regression analyses for comparison of BMD and BMAD at the hip, spine and whole-body among sarcopenia and obesity groups

|  | **Obesity vs *Controls*** | **Sarcopenia vs *Controls*** | **Sarcopenic obesity vs *Controls*** | **Sarcopenic obesity vs *Obesity*** | **Sarcopenic Obesity vs *Sarcopenia*** |
| --- | --- | --- | --- | --- | --- |
| Hip BMD  Model 1  Model 2  Model 3  Spine BMD  Model 1  Model 2  Model 3  Whole-body BMD  Model 1  Model 2  Model 3  Hip BMAD  Model 1  Model 2  Model 3  Spine BMAD  Model 1  Model 2  Model 3  Whole-body BMAD  Model 1  Model 2  Model 3 | 0.019 (-0.002, 0.040)  **0.025 (0.007, 0.043)**  **0.024 (0.005, 0.043)**  0.007 (-0.025, 0.039)  0.025 (-0.005, 0.054)  0.028 (-0.003, 0.059)  **-0.061 (-0.082, -0.039)**  **-0.049 (-0.070, -0.028)**  **-0.039 (-0.061, -0.017)**  **0.006 (0.002, 0.009)**  **0.005 (0.001, 0.008)**  **0.004 (0.000, 0.007)**  0.002 (-0.001, 0.006)  0.003 (-0.001, 0.007)  0.003 (-0.001, 0.007)  **-0.002 (-0.002, -0.001)**  **-0.002 (-0.002, -0.001)**  **-0.001 (-0.002, 0.000)** | **-0.058 (-0.077, -0.038)**  **-0.066 (-0.083, -0.049)**  **-0.065 (-0.082, -0.048)**  0.014 (-0.016, 0.043)  -0.022 (-0.050, 0.006)  -0.024 (-0.053, 0.004)  -0.018 (-0.038, 0.002)  **-0.034 (-0.054, -0.013)**  **-0.035 (-0.056, -0.015)**  **-0.011 (-0.014, -0.008)**  **-0.008 (-0.011, -0.005)**  **-0.008 (-0.011, -0.004)**  0.001 (-0.002, 0.005)  0.000 (-0.004, 0.004)  0.000 (-0.003, 0.004)  0.000 (0.000, 0.001)  0.000 (0.000, 0.001)  0.000 (0.000, 0.001) | **-0.057 (-0.094, -0.019)**  **-0.059 (-0.091, -0.028)**  **-0.059 (-0.090, -0.028)**  -0.040 (-0.097, 0.016)  **-0.060 (-0.111, -0.009)**  **-0.061 (-0.112, -0.009)**  **-0.082 (-0.120, -0.044)**  **-0.087 (-0.123, -0.050)**  **-0.083 (-0.119, -0.046)**  **-0.007 (-0.013, 0.000)**  -0.004 (-0.010, 0.002)  -0.004 (-0.010, 0.001)  -0.004 (-0.011, 0.003)  -0.004 (-0.011, 0.002)  -0.004 (-0.011, 0.002)  **-0.001 (-0.002, -0.001)**  **-0.001 (-0.002, -0.001)**  **-0.001 (-0.002, -0.001)** | **-0.076 (-0.115, -0.037)**  **-0.084 (-0.117, -0.052)**  **-0.083 (-0.111, -0.067)**  -0.047 (-0.106, 0.012)  **-0.085 (-0.139, -0.031)**  **-0.089 (-0.143, -0.035)**  -0.021 (-0.061, 0.019)  -0.038 (-0.076, 0.001)  **-0.044 (-0.083, -0.006)**  **-0.012 (-0.018, -0.006)**  **-0.009 (-0.015, -0.003)**  **-0.008 (-0.015, -0.002)**  -0.006 (-0.013, 0.000)  **-0.007 (-0.014, 0.000)**  **-0.007 (-0.014, 0.000)**  0.000 (-0.001, 0.001)  0.000 (-0.001, 0.001)  0.000 (-0.001, 0.001) | 0.001 (-0.037, 0.039)  0.006 (-0.025, 0.038)  0.006 (-0.026, 0.038)  -0.054 (-0.112, 0.004)  -0.039 (-0.090, 0.013)  -0.036 (-0.089, 0.016)  **-0.064 (-0.103, -0.025)**  **-0.053 (-0.090, -0.016)**  **-0.047 (-0.085, -0.010)**  0.004 (-0.002, 0.010)  0.004 (-0.003, 0.010)  0.003 (-0.003, 0.009)  -0.005 (-0.012, 0.002)  -0.004 (-0.011, 0.002)  -0.004 (-0.011, 0.002)  **-0.002 (-0.003, -0.001)**  **-0.002 (-0.002, -0.001)**  **-0.001 (-0.002, -0.001)** |

Data presented as **β**-coefficients and 95% confidence intervals. Bold indicated p<0.05. Abbreviations: O, obesity; S, sarcopenia; SO, sarcopenic obesity; BMD, bone mineral density; BMAD, bone mineral apparent density

Model 1: Unadjusted model

Model 2: Adjusted for confounders including age, smoking status and protein intake

Model 3: Adjusted for confounders in model 2 and socioeconomic status including education and occupation levels
